# Intraperitoneal injection of Desferal® alleviated the age-related bone loss and senescence of bone marrow stromal cells in rats

**DOI:** 10.1186/s13287-020-02112-9

**Published:** 2021-01-07

**Authors:** Lingxian Yi, Yue Ju, Ying He, Xiushan Yin, Ye Xu, Tujun Weng

**Affiliations:** 1 Department of Orthopaedics, Fourth medical center of PLA General Hospital, No. 51 Fucheng Road, Beijing, 10048 People’s Republic of China; 2 Critical Care Medicine Department, PLA Strategic Support Force Characteristic Medical Center, Beijing, 100101 China; 3 Applied Biology Laboratory, Shenyang University of Chemical Technology, Shenyang, China; 4 School of Mechanical Engineering and Automation, Beihang University, Beijing, People’s Republic of China

**Keywords:** Bone loss, Bone marrow stromal cells, Hypoxia, Aging, Desferal®

## Abstract

**Background:**

Age-related bone loss plays a vital role in the development of osteoporosis and osteoporotic fracture. Bone marrow stromal cell (BMSC) senescence is highly associated with osteoporosis and limits the application of BMSCs in regenerative medicine. Hypoxia is an essential component for maintaining the normal physiology of BMSCs. We have reported that activation of hypoxia-induced factor by deletion of von Hippel-Lindau gene in osteochondral progenitor cells protected mice from aging-induced bone loss. However, whether pharmacologically manipulation of hypoxic niche would attenuate age-related bone loss and dysfunction of BMSCs is not well understood.

**Methods:**

Twelve-month-old Sprague-Dawley rats were used as an aged model and were intraperitoneally injected with Desferal® (20, 60 mg/kg weight or vehicle), three times a week for a continuous 8-week period. Two-month-old young rats were set as a reference. After 8 weeks, micro-CT and HE staining were performed to determine the effect of Desferal® on bone loss. In order to investigate the effects of Desferal® on BMSC senescence, 12-month-old rats were treated with high-dose Desferal® (60 mg/kg weight) daily for 10 days. BMSCs were isolated and evaluated using CCK-8 assay, colony-forming cell assay, cell differentiation assay, laser confocal for reactive oxygen species (ROS) level, senescence-associated β-galactosidase (SA-β-gal) staining, and molecular expression test for stemness/senescence-associated genes.

**Results:**

Micro-CT and HE staining showed that high-dose Desferal® significantly prevented bone loss in aged rats. Compared with vehicle group, the ex vivo experiments showed that short-term Desferal® administration could promote the potential of BMSC growth (proliferation and colony formation ability) and improve the rebalance of osteogenic and adipogenic differentiation, as well as rejuvenate senescent BMSCs (ROS level and SA-β-gal staining) and revise the expression of stemness/senescence-associated genes. The potential of BMSCs from 12M-H-Desferal® group at least partly revised to the level close to 2-month-old group.

**Conclusions:**

The current study suggested that Desferal®, an iron-chelating agent, could alleviate age-related bone loss in middle-aged rats. Meanwhile, we found that short-term intraperitoneal injection of Desferal® partly rejuvenate BMSCs from aged rats. Overall, we demonstrated a novel role of Desferal® in rejuvenating aged BMSCs and preventing age-related bone loss.

**Supplementary Information:**

The online version contains supplementary material available at 10.1186/s13287-020-02112-9.

## Background

Age-related bone loss has been well recognized in both human and animal. It is well understood that aging is accompanied by a gradual decline in bone mass, bone quality, and strength, which cooperatively increase the risk of fractures. As a result, osteoporosis and osteoporotic fracture were recognized as major public problems for the elderly [1].

Bone marrow stromal cells (BMSCs) exist in the bone marrow with multi-potency and have a broad application prospect in the field of cell therapy and regenerative medicine thanks to their accessibility and expansion potential [2]. Previous study showed a high potential association between BMSC senescence and age-related bone loss [3]. Several studies have documented that age drives the intrinsic alterations of BMSCs, including decreased proliferation and osteogenic differentiation potential, as well as increased senescence-associated gene expression and β-galactosidase-positive staining [4, 5]. It also reported that the viability of aged BMSCs decreased, and senescent BMSCs were more likely to differentiate into adipocytes [6]. These changes led to the decrease in quantity and quality of BMSCs, which together contributed to age-related bone loss. In addition, dysfunction of BMSCs from elders limited their application in the field of cell therapy and regenerative medicine [7]. Therefore, delaying or rejuvenating the senescence of BMSCs is of great significance for the treatment of osteoporosis and the application of regenerative medicine.

Oxygen is a fundamental element of the bone marrow niche, and a hypoxic environment in the bone marrow is generally considered to be indispensable for retaining normal physiological function and self-renewal of stromal cells [8]. BMSCs cultured under hypoxic conditions may be useful for enhancing cell proliferation, viability, and colony formation [9]. It has been reported that serial passage in the culture of MSCs at an oxygen concentration of 1% can inhibit cellular senescence by reducing the expression of p16 and extracellular signaling regulated kinase [10]. As the key transcription factor response to hypoxia stress, hypoxia-induced factor 1α (HIF-1α) is a highly unstable protein in normoxic conditions. However, under hypoxic conditions, the catalytic activity of prolyl hydroxylases (PHD) is inhibited, leading to the stabilized expression of HIF-1α [11]. Some small molecules, such as deferoxamine (DFO), are known as hypoxia mimics, which can elevate HIF-1α levels by blocking PHD activity even in normoxic conditions [12]. In bone, the HIF-1α has been shown to modulate the recruitment and function of osteoblast and osteoclast precursors at sites of injury [13]. Zhao et al. further found that activation of the HIF-1α pathway in osteoblasts by genetic methods or DFO treatment would protect mice from ovariectomy-induced osteoporosis by targeting the coupling of angiogenesis and osteogenesis [14]. Previously, we also found that activating the HIF pathway in bone progenitor cells by deletion of the von Hippel Lindau gene antagonizes age-related bone loss [15]. In addition, some studies suggested that pretreating with hypoxia or hypoxia mimics improved the osteogenic and angiogenic ability of BMSCs and enhanced bone repair in bone tissue engineering [16, 17]. Based on those findings, we propose the hypothesis that pharmacological manipulation of a hypoxic niche would attenuate age-related bone loss and rejuvenate the senescence of BMSCs.

In this study, Desferal®, deferoxamine mesylate for injection, which is approved for the treatment of acute iron intoxication and chronic iron overload, was used to explore the beneficial effects on preventing aging-induced bone loss and mitigating dysfunction of BMSCs from elders. Following the injection with Desferal® or vehicle intraperitoneally, vertebras and femurs from 12-month-old rats were used to determine the bone mass by histological and micro-CT analysis. In addition, BMSCs were isolated from bone marrow to evaluate the age-related characteristics in vitro. Our results showed that Desferal® could partly prevent age-related bone loss and mitigate the dysfunction of BMSCs from elders.

## Methods

### Animal model and study design

Male Sprague-Dawley (SD) rats were used in all of the experiments described. The use of laboratory rats and experimental animal procedures was approved by the Experimental Animals Center of the Fourth medical center of PLA general hospital. The SD rats arrived at our animal center 1 week before the experiment, and the facility is temperature-, ventilation-, and illumination-controlled. The animals had access to food pellets and tap water. For histological analysis, a total of 25 rats were divided into four groups. Six 2-month-old rats were set as reference group (2M group), and nineteen 12-month-old rats were randomly divided into three groups according to the injected doses of Desferal®: the vehicle group which received saline (*n* = 6, 12M-VEH group), the high-dose Desferal® group (*n* = 7, 60 mg/kg weight, 12M-H-Desferal®), and the low-dose Desferal® group (*n* = 6, 20 mg/kg weight, 12M-L-Desferal®). All of rats in 12-month-old groups were intraperitoneally injected with different doses of Desferal® or vehicle three times a week for 8 weeks. Rats could move freely in their cages and get enough food and water ad libitum. For ex vivo cell analysis, rats were divided into three groups and were subjected to short-term injection with Desferal® or saline. The first two groups of 12-month-old rats were continuously intraperitoneally injected with Desferal® (60 mg/kg) or vehicle, respectively, once a day for ten consecutive days. The third group consisting of 2-month-old rats was served as the positive control. Rats were then killed with carbon dioxide, and the long bones including femurs and tibias were dissected and separated to isolate BMSCs. Two rats were used in each group every time and repeated for three times.

### Analysis of micro-CT scans

After 8 weeks of administration, femurs from rats of all groups were harvested and fixed with 4% paraformaldehyde. All left femur of each group were scanned with a Scanco vivaCT 40 instrument (Scanco, Brüttisellen, Switzerland) and analyzed for bone structure. Briefly, serial 17.5-μm tomographic images were acquired at 70 kV and 113 mA and 3D volume images were reconstructed using the provided micro-CT software. The region of interest (ROI) in the trabecular bone started at 100 slices (1750 μm) below the lowest point of the growth plate and the range was for 100 slices (1750 μm). A constant threshold (190) was set to segment trabecular bone from bone marrow. Trabecular morphometry parameters including bone volume fraction (bone volume (BV)/ total volume (TV)), trabecular thickness (Tb.Th), trabecular number (Tb.N), trabecular separation (Tb.Sp), trabecular connectivity density (Conn.D), and structural model index (SMI) were measured.

### Histological evaluation

The animals were euthanized by CO_2_ overdose at 8 weeks postinjection, and the vertebras and femurs were collected for analysis. For histological analysis, vertebras were fixed in 4% paraformaldehyde in 0.1 M phosphate buffer for 48 h and subsequently decalcified in 15% ethylenediaminetetraacetic acid (EDTA) solution for 3 weeks before being embedded in paraffin. Sections with thicknesses of 5 μm were cut and stained with hematoxylin/eosin (HE) staining according to standard methods. The images of vertebra trabeculae were acquired with a light microscope (Olympus, Japan) and Nikon digital imaging system. The Bone Histomorphometry System OsteoMeasure (OsteoMetrics, Inc.) was used to calculate the percent area of mineralized bone, fatty marrow, and red marrow in the region of interest (ROI). The ROIs for three compartments data collection were measured in an area of 2.0 mm in length from 1 mm below the endplate of the vertebras.

### Rat bone marrow stromal cells isolation and cultivation

Bone stromal cells were isolated from the limb long bones of SD rats including femurs and tibias with a rat bone marrow stem cell isolation kit (TBD Science, China) according to the instructions under aseptic condition. Briefly, after euthanasia, long bones were dissected free of soft tissues and cut open at both ends with scissors. Then bone marrow was flushed out from bone cavity with rinsing solution provided in the kit using a 22-gauge syringe. Bone marrow was blown repeatedly to produce a single-cell suspension, followed by filtration through a 70-μm nylon mesh filter. After being centrifuged at 450×*g* for 10 min, the supernatant of the single-cell suspension was discarded. Cell pellets were suspended with the sample dilution. Finally, the primary cells were seeded at a density of 1.5–3× 10^6^ /ml on *10*-*cm* dishes with the serum-free stem-cell culture medium (TBD Science, China) in a humidified atmosphere with 5% CO_2_ at 37 °C. The non-adherent cells were removed by changing the culture medium. When the adherent cells grew to 85–95% confluence, they were digested with trypsin and collected for follow-up experiments.

### Cell proliferation assay

Cell proliferation was determined with the Cell-Counting Kit-8 (CCK-8; Meilunbio, China) following the manufacturer’s instructions. Briefly, BMSCs at passage 2 were seeded at a density of 1 × 10^3^ cells per well in a 96-well plate at 37 °C with 5% CO_2_. Once the cells adhered to the plate, CCK-8 solution was added. After incubation for 1 h at 37 °C, a microplate reader (Thermo Scientific Multiskan FC, USA) was used to examine the absorbance of the solution at 450 nm. Cell proliferation was measured for three consecutive days. All assays were carried out in triplicate. A histogram was made based on the absorbance values.

### Colony-forming assay

Primary BMSCs were counted with a hemocytometer. Appropriate 3 × 10^3^ BMSCs were plated in a 6-well plate and cultured with 3 ml complete medium. The cells were uniformly dispersed and then routinely cultured for colony formation. After 14 days of culture, the clones were stained with toluidine blue and the images were obtained by scanning the culture plate. For colony quantification, 5 × 10^3^ BMSCs were plated in a T25 culture flask and cultured for 14 days. Only aggregates with more than 50 cells was defined as a colony and included in the counting. The experiment was performed in duplicate and repeated twice.

### Cell differentiation assay

For osteogenic differentiation, the BMSCs at passage 2 were cultured at a density of 5 × 10^4^ cells/well in a 24-well plate. When cells reached confluence, the growth medium was replaced by osteogenic differentiation medium, that is complete medium supplied with 0.05 mM ascorbate-2-phosphate (Sigma-Aldrich, USA) and 10 mM β-glycerophosphate (Sigma-Aldrich, USA) for 7 or 18 days; afterward, the cells were fixed with 4% PFA and subjected to alkaline phosphatase (ALP; Beyotime, China) or alizarin red S staining (Sigma-Aldrich, USA). For adipogenic differentiation, the BMSCs at passage 2 were cultured at a density of 5 × 10^4^ cells/well in a 24-well plate and treated with OriCell adipogenic differentiation medium (Cyagen, China) for 21 days and finally stained with Oil red-O according to the provided instructions. For quantification of adipogenic difference, the percentage of Oil red-O positive cells within all cells for each group was quantified from five random images per well using OsteoMeasure software (OsteoMetrics, Inc.).

The ALP activity was determined with Alkaline Phosphatase Assay Kit (Beyotime, China) according to the manufacturer’s instructions. Briefly, BMSCs in a 24-well plate were osteoblastic differentiation for 7 days, and then were lysed with 100 μl of cell lysis buffer without inhibitor (Beyotime, China). The absorbance value of the sample was calculated by detecting the absorbance value of at the wavelength of 405 nm, and the ALP enzyme activity was calculated according to the standard curve. For quantification of matrix calcification, the alizarin red S-stained mineralized nodules were desorbed with 10% cetylpyridinium chloride (Sigma-Aldrich, USA) and the OD value was measured at the wavelength of 415 nm.

### Senescence-associated β-galactosidase (SA-β-gal) staining

The senescence of BMSCs was determined by SA-β-gal staining kit (Beyotime, Shanghai, China). Briefly, BMSCs at passage 2 were plated at a density of 5 × 10^4^ /well in a 12-well plate. When reached 40% confluence, the cells were fixed with 4% paraformaldehyde at room temperature for 20 min and subsequently stained with the commercial kit according to the manufacturer’s instructions. Senescent cells were stained by the blue color precipitate over the cell, and the images were photographed by a light microscope and digital imaging system (Olympus, Japan). For quantification of BMSC senescence, the percentage of SA-β-gal-positive cells normalized to total cell number for each group was quantified from five random images per well using OsteoMeasure software (OsteoMetrics, Inc.).

### Reactive oxygen species (ROS) generation assay

The intracellular ROS levels were analyzed by ROS assay kit (Beyotime, Shanghai, China) following the manufacturer’s introductions. Briefly, 5 × 10^4^ BMSCs (passage 2) were grown on a glass coverslip in a 24-well plate and cultured for 24 h in a humidified 5% CO_2_ environment at 37 °C. Then the cells were treated with 25 mM 2′,7′-dichlorofluorescein diacetate (DCFH-DA) at 37 °C for 30 min. ROS reacts with a fluorescence sensor located in the cytoplasm to produce a fluorescent product named as 2′, 7′-dichlorodihydrofluorescein (DCF), which is proportional to the content of ROS. Rosup treatment was a positive control. DAPI staining used to visualize cell nuclear. The fluorescence signal of cells in each group was detected at 488-nm excitation and 525-nm emission wavelengths by a laser confocal microscope (Zeiss, Germany), respectively. The representative images were acquired by photo stitching from randomly selected four adjacent fields at a magnification of × 200. For quantification of ROS production, fluorescence intensity of BMSCs was determined by microplate assays on a 96-well plate using ROS assay kit. Briefly, BMSCs at passage 3 were seeded on the 96-well plate for 5000 cells each well and the ROS generation were measured with microplate reader at 488 nm according the procedure described above.

### Real-time polymerase chain reaction

Total RNA was extracted from cells of each group with TRIzol Reagent (Invitrogen) following the manufacturer’s instructions. Then RNA (1 μg) was reverse-transcribed into cDNA with All-in-One cDNA Synthesis SuperMix (Bimake, USA). Real-time PCR was performed to determine the relative mRNA levels using the QuantStudio 5 (Applied Biosystem, USA) and UltraSYBR Mixture (Cwbio, China). Each sample was run in triplicate. GAPDH was used as an internal control to evaluate the relative expression. Cycling parameters are as follows: activation of heat-activated DNA polymerase at 95 °C for 5 min, amplification for 40 cycles (denaturation at 95 °C, 30 s; annealing at 60 °C, 30 s; extension at 72 °C, 30 s), melt curve for 1 cycle (95 °C, 15 s; 60 °C, 60 s; 95 °C, 15 s). Finally, gene expression was obtained using QuantStudio Design & Analysis desktop software. The primers were designed with the Primer Premier 5 software and presented as follows: GAPDH Forward, 5′- CGT ATC GGA CGC CTG GTT A-3′ and Reverse, 5′-TCG CTC CTG GAA GAT GGT G-3′; p16 Forward, 5′-GGG TCA CCG ACA GGC ATA A-3′ and Reverse, 5′-TCT CGC GTT GCC AGA AGT G-3′; p21 Forward, 5′- CAC AGG AGC AAA GTA TGC CGT C-3′ and Reverse, 5′-GCG AAG TCA AAG TTC CAC CGT-3′; p53 Forward, 5′- CAG ATT GGG GAA TGG GTT GG-3′ and Reverse, 5′-GCA GAG TGG AGG AAA TGG GTC-3′; Nanog Forward, 5′- TCC TCA CCA AGA AAG CAG AAG AT-3′ and Reverse, 5′-GCT CAG GCT CAG AAT GGT AGA GA-3′; OCT-4 Forward, 5′- TCT ACT CGG TCC CTT TTC CTG A-3′ and Reverse, 5′-TTT GTC TAC CTC CCT TCC TTG C-3′.

### Western blot analysis

Proteins were lysed from BMSCs in a 6-well plate using a RIPA lysis buffer containing protease inhibitors (Boster Biological Technology co.ltd, China). After measurement of the protein concentration with BCA protein assay kit (Boster Biological Technology co.ltd, China), proteins of the same quality (30 μg) were separated on sodium dodecyl sulfate-polyacrylamide electrophoresis gels and transferred to 0.45 μm PVDF membranes (Millipore). The membranes were blocked with 5% milk resolved in Tris buffered saline-Tween buffer, and probed by diluted antibodies. Primary antibodies used included the following: Hif1α (Boster Biological Technology co.ltd, China), P16 (Abcam, UK), p21 (Boster Biological Technology co.ltd, China), p53 (Cell Signaling Technology, USA), and GAPDH (Proteintech Group, Inc., USA). The grayscale of the band were quantified using ImageJ software. The relative value of the target gene expression was obtained by dividing the gray value of the target gene by the gray value of the internal reference band (GAPDH).

### Statistical analysis

All results are expressed as means ± standard deviation. Statistical analysis was performed using SPSS software (version 20, IBM, NY, USA). One-way ANOVA and Dunn’s multiple comparison test were employed to determine the significant difference, and *p* < 0.05 was considered statistically.

## Results

### The effect of long-term Desferal® treatment on age-related bone loss in middle-aged rats

**Fig. 1:**
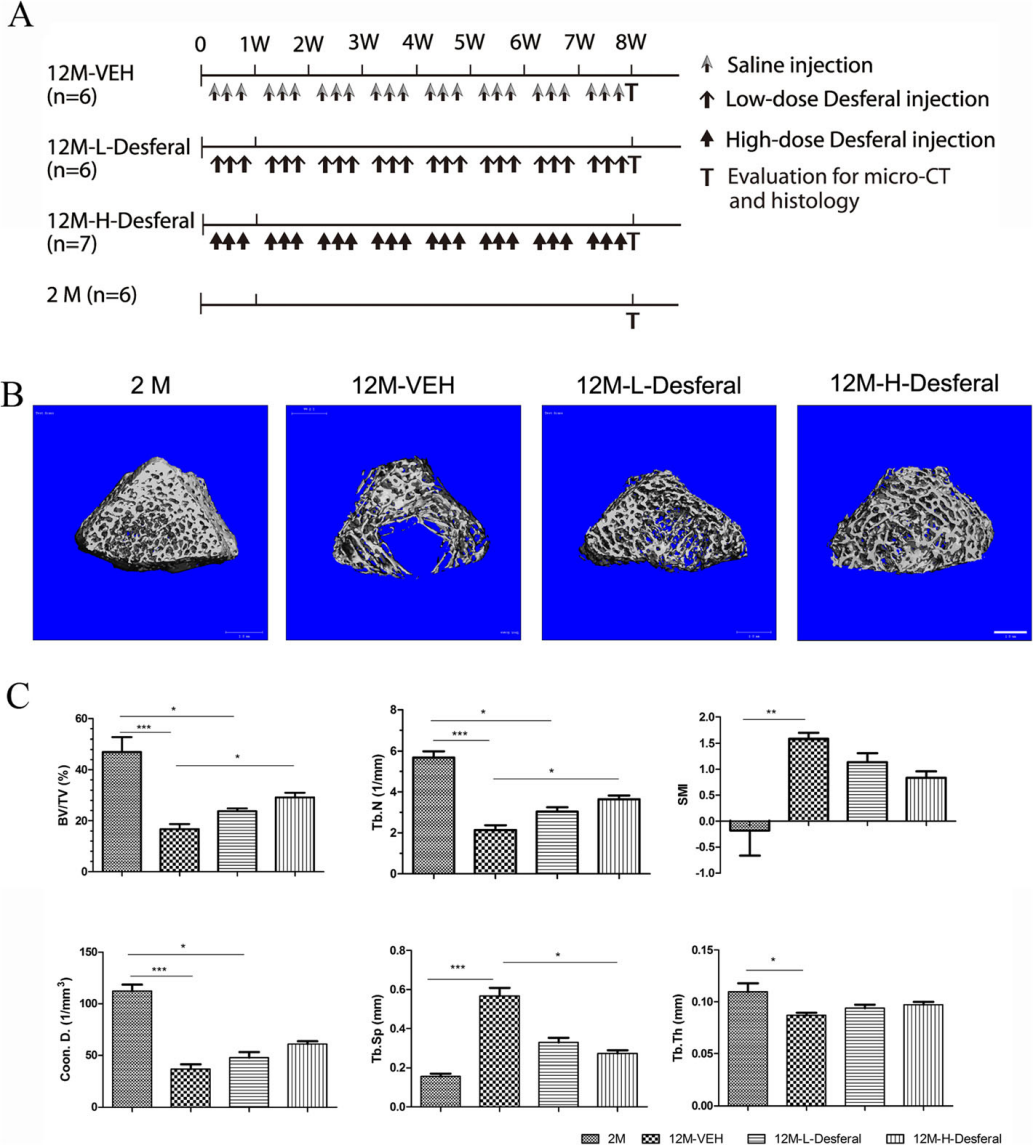
micro-CT analysis of rat femur following intraperitoneal injection of Desferal®. **a** Schema of long-term Desferal administration of the in vivo study. **b** Representative three-dimensional micro-CT images of femurs in 2M, 12M-VEH, 12M-L-Desferal® (20 mg/kg), and 12M-H-Desferal® (60 mg/kg) groups. Bar = 1 mm. **c** Quantification of bone morphometric parameters of rat femur by micro-CT analysis. Significantly increased BV/TV, Tb.N and Coon.D and decreased Tb.Sp and SMI were observed in the 12M-H-Desferal® group when related to those of the 12M-VEH group. All of these parameters were statistically significant between the 2M and 12M-VEH groups. After Desferal® treatment, especially in the 12M-H-Desferal® group, bone mass increased significantly.BV/TV, bone volume/total volume; Tb.N, trabecular number; Tb.Th, trabecular thickness; Coon.D, trabecular connectivity density; Tb.Sp, trabecular separation; SMI, structural model index. *p < 0.05, **p < 0.01, ***p < 0.001

**Fig. 2:**
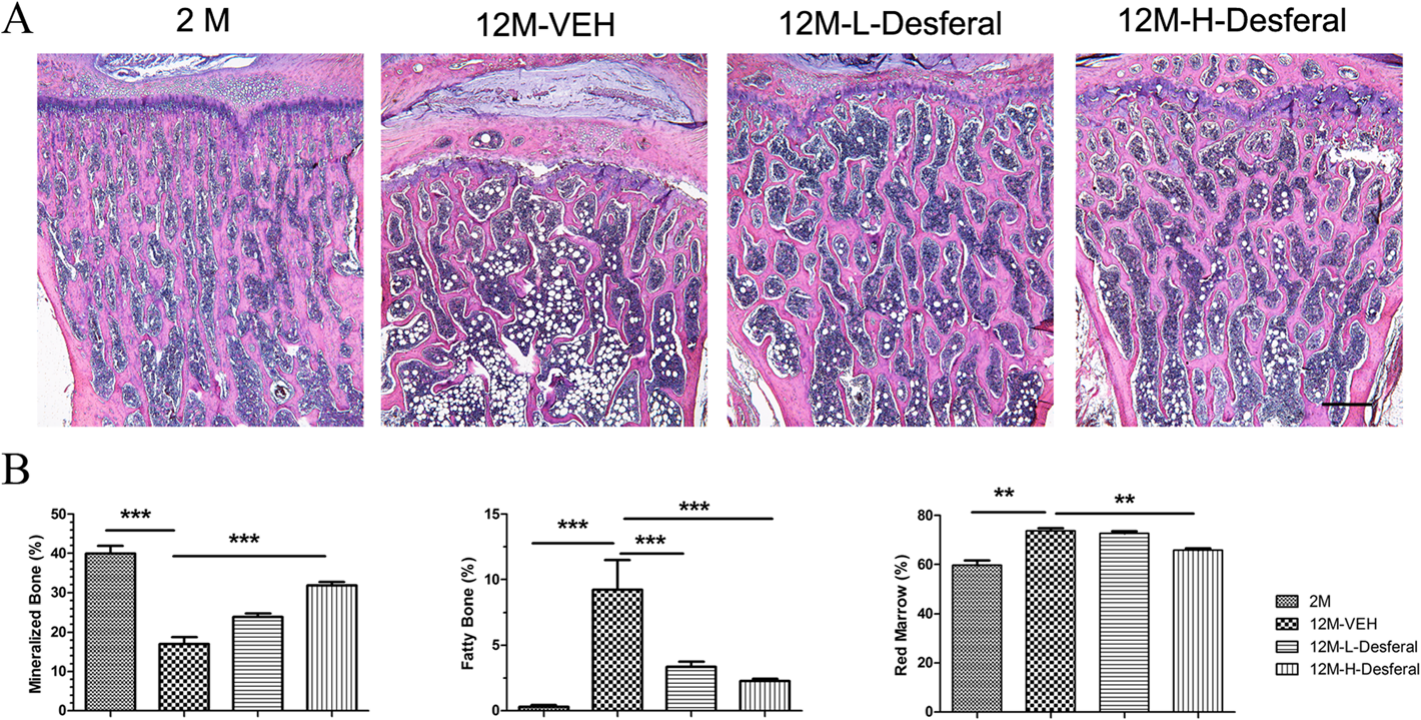
Histological appearance of rats vertebras following Desferal® treatment. **a** Hematoxylin/eosin (HE) staining images of vertebras. Bar = 500 μm. **b** Quantitative assessment the percent area of mineralized bone, fatty marrow and red marrow in the region of interest, which was an area 2 mm in length from 1 mm below the endplate of the vertebras. Followed by Desferal® treatment, especially in the 12M-H-Desferal® group, bone mass was increased significantly and adipocyte numbers were reduced. **p < 0.01, ***p < 0.001

To determine the effect of Desferal® on aging-related bone loss, we administered an intraperitoneal injection of Desferal® with a high or low dose in an aged model to compare the osteoprotective effect to that from vehicle (Fig. 1a). Femurs and vertebras were harvested from four groups and performed with micro-CT and histological analysis, respectively. From 3D micro-CT reconstructed images, dramatically decreased trabecular bone was observed in the 12M-VEH group when compared to 2M group, and a dose-dependent increase of trabecular bone was found after injection with Desferal® (Fig. 1b). Quantification of trabecular bone in femur showed significant reductions in BV/TV, Tb.N, Tb.Th, and Coon.D, as well as increased Tb.Sp and SMI in 12M-VEH group compared with those in 2M group (Fig. 1c). Desferal® treatment, especially at high doses group (60 mg/kg), significantly reduced the deterioration in bone microarchitecture induced by aging and increased the trabecular bone mass for near 50% following 8 weeks’ treatment (Fig. 1c). Consistently, similar results were also found in vertebras by HE staining (Fig. 2a). Notably, more adipocytes were observed in the 12M-VEH group while the percent area of fatty marrow was significantly decreased after Desferal® treatment (Fig. 2a, b). Histomorphometric analysis suggested that the amount of mineralized bone was severely decreased during the aging process, while long-term Desferal® treatment significantly alleviated age-related bone loss. Our results indicated that long-term treatment of Desferal®, a hypoxic mimetic agent, may have a potential preventive effect on age-related bone loss.

### Short-term Desferal® treatment promoted cell growth and relieved the imbalance of osteogenic and adipogenic differentiation in middle-aged BMSCs

**Fig. 3:**
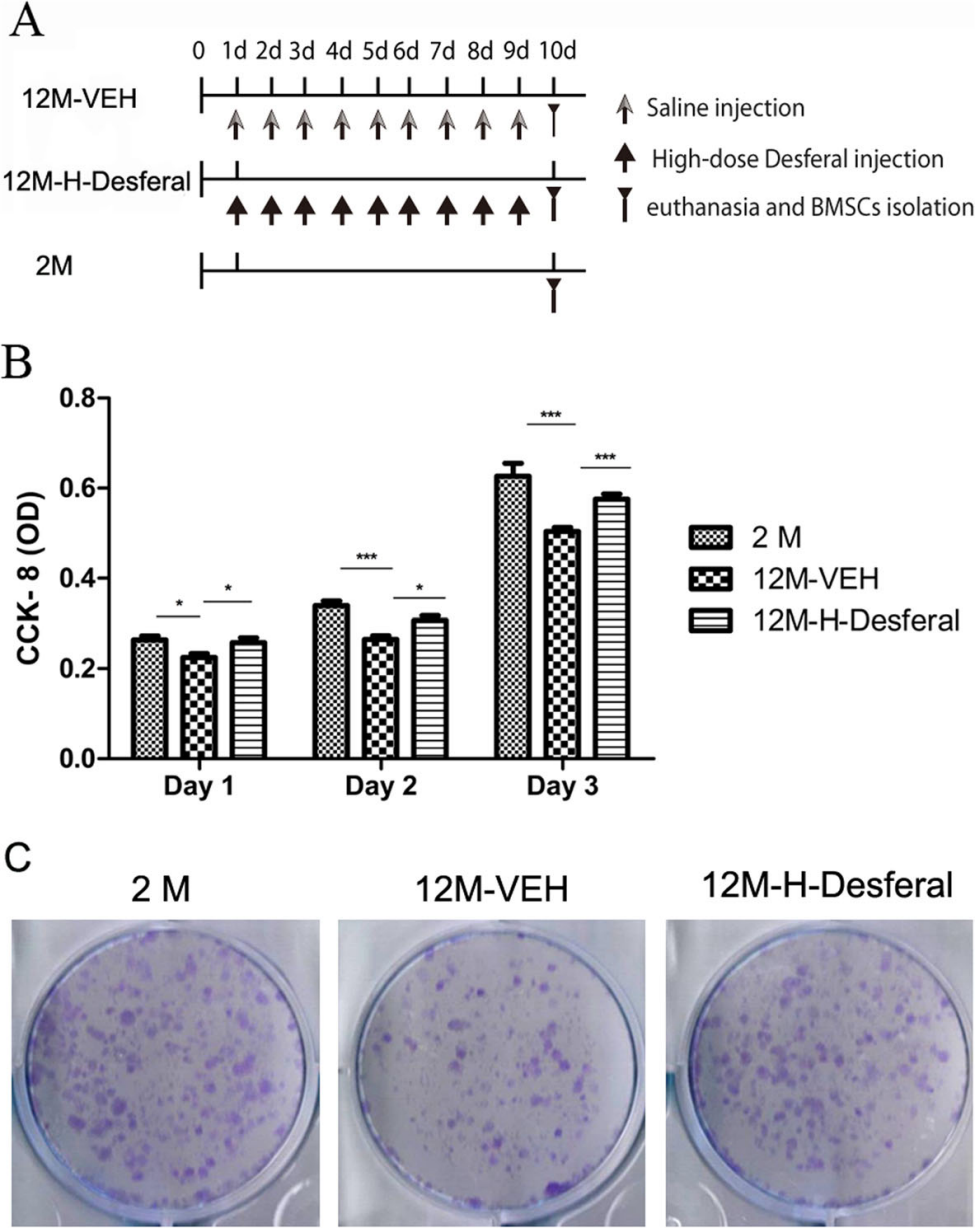
Cell proliferation and colony-forming of BMSCs from 2M, 12M-VEH and 12M-H-Desferal group rats. **a** Schema of short-term Desferal® administration of the ex vivo study. **b** CCK-8 assay of cell proliferation. The optical density (OD) value was measured at 450 nm absorbance. In BMSCs from 12M-H-Desferal® group, the proliferation rate (OD value) significantly increased compared with those from 12M-VEH group. While the OD values of 12M-VEH group are lower than those from 2M group at day 1–3. The data were drawn from three independent experiments and the results were expressed as mean ± SD. *p < 0.05, ***p < 0.001. **c** Colony-forming cell assay. Decreased cell colonies in BMSCs from the 12M-VEH group compared with that from the 2M group. While cell colonies in BMSCs from the 12M-H-Desferal® group increased significantly compared with that from the 12M-VEH group

In addition to the results showing long-term effect of Desferal® injection, we investigated the changes of cell characteristic of BMSCs in the bone marrow exposure to short-term Desferal® treatment. BMSCs from 12-month-old rats following short-term Desferal® treatment were harvested and analyzed (Fig. 3a). CCK-8 assay and colony formation were performed to determine the cell potential of BMSCs from rats treated with and without Desferal® daily for 10 days. In line with previous studies, BMSCs from middle-aged rats showed a strong reduction in cell proliferation and colony formation (Fig. 3b,c). As shown in Fig. 3b, compared with BMSCs from 12M-VEH rats, the proliferation potential of BMSCs from 12M-H-Desferal® group was significantly increased from day 1 to day 3. Meanwhile, colony formation assay was also used to evaluate cell viability. The result of toluidine-blue staining showed that Desferal® treatment dramatically restored the clone formation capability of senescent BMSCs in ex vivo experiment (Fig. 3c). Quantification of colony-forming assay in T25 culture flask also showed the number of clones (> 50 cells) in the 12M-H-Desferal® group was significantly increased compared with those in 12M-VEH group (Fig. S2). These results suggested that short-term Desferal® treatment could promote the potential of BMSCs proliferation in the middle-aged rat.

**Fig. 4:**
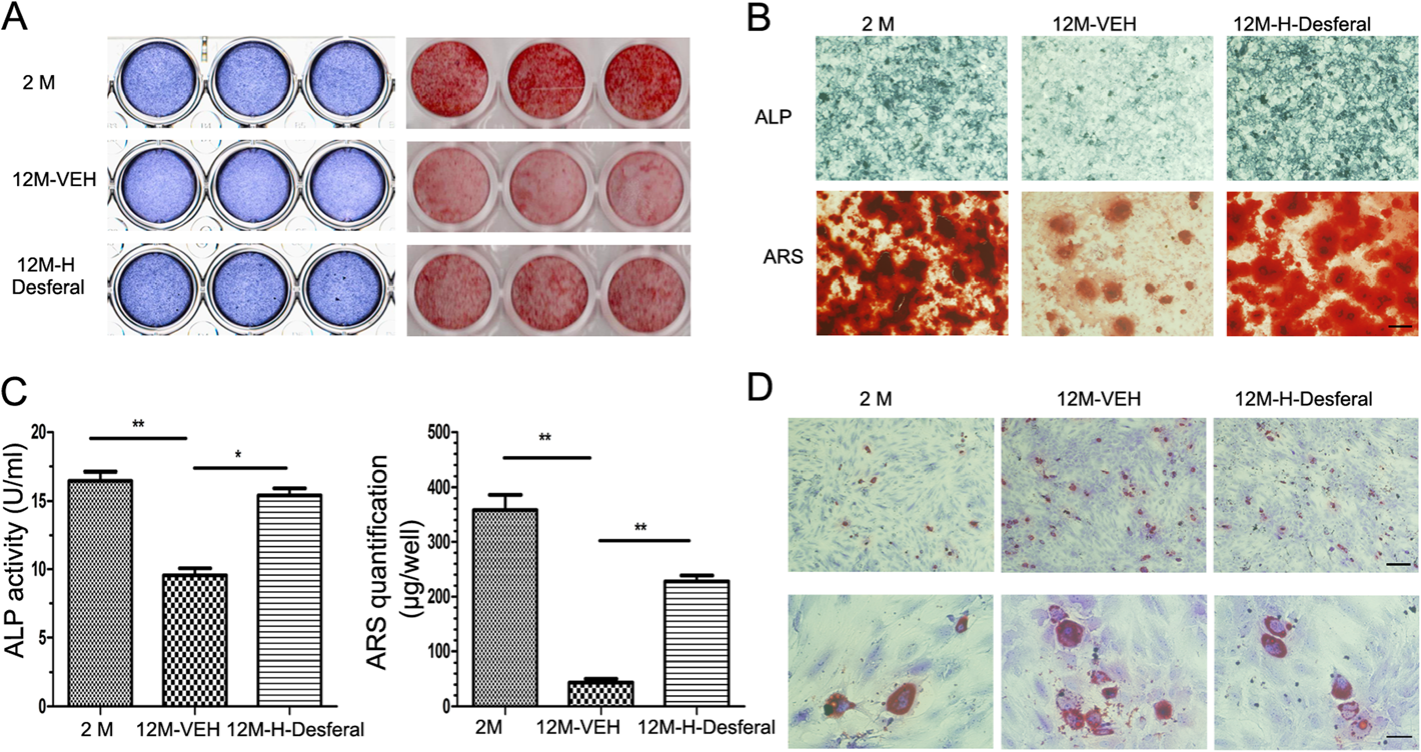
Osteogenic and adipogenic differentiation of BMSCs from rats with and without Desferal® treatment. **a** Osteogenic differentiation was determined by ALP staining at day 7 and Alizarin Red S staining at day 18 after osteogenic induction. Scanned images from a 24-well plate. **b** Higher magnification images of ALP and Alizarin Red S staining. Bar=500 μm. **c** ALP activities of BMSCs after osteogenic differentiation for 7 days and quantitative analysis of Alizarin Red S staining at day 18. *p < 0.05, **p < 0.01. **d** Oil red-O staining for adipocytes after adipogenic differentiation for 21 days. Top bar = 500 μm, bottom bar = 100 μm

Based on the results that osteogenic and adipogenic differentiation potential of BMSCs were markedly changed during aging, the effects on the balance of osteogenic and adipogenic differentiation of BMSCs from Desferal®-treated rats were investigated. Consistent with previous studies, the osteogenic differentiation potential of BMSCs from the 12M-VEH group was significantly decreased compared with those from 2M group rats, and short-term Desferal® treatment improved osteogenic differentiation of aged BMSC (Fig. 4a, b). ALP activity assay and quantification of Alizarin red staining also suggested that BMSCs from 12M-Desferal® group showed an increase in osteogenic differentiation and calcium deposition (Fig. 4c). Consistent with the results of the histological analysis, the adipogenic assay revealed a striking increase in the number of adipocytes in BMSCs from the 12M-VEH group (Fig. 4d). In addition, short-term Desferal® treatment suppressed the adipogenic differentiation potential of BMSCs (Fig. 4d, Fig. S3). These results suggested that short-term Desferal® treatment could improve the rebalance of osteogenic and adipogenic differentiation in BMSCs from middle-aged rats.

### Short-term Desferal® treatment inhibited cell senescence in middle-aged BMSCs

**Fig. 5:**
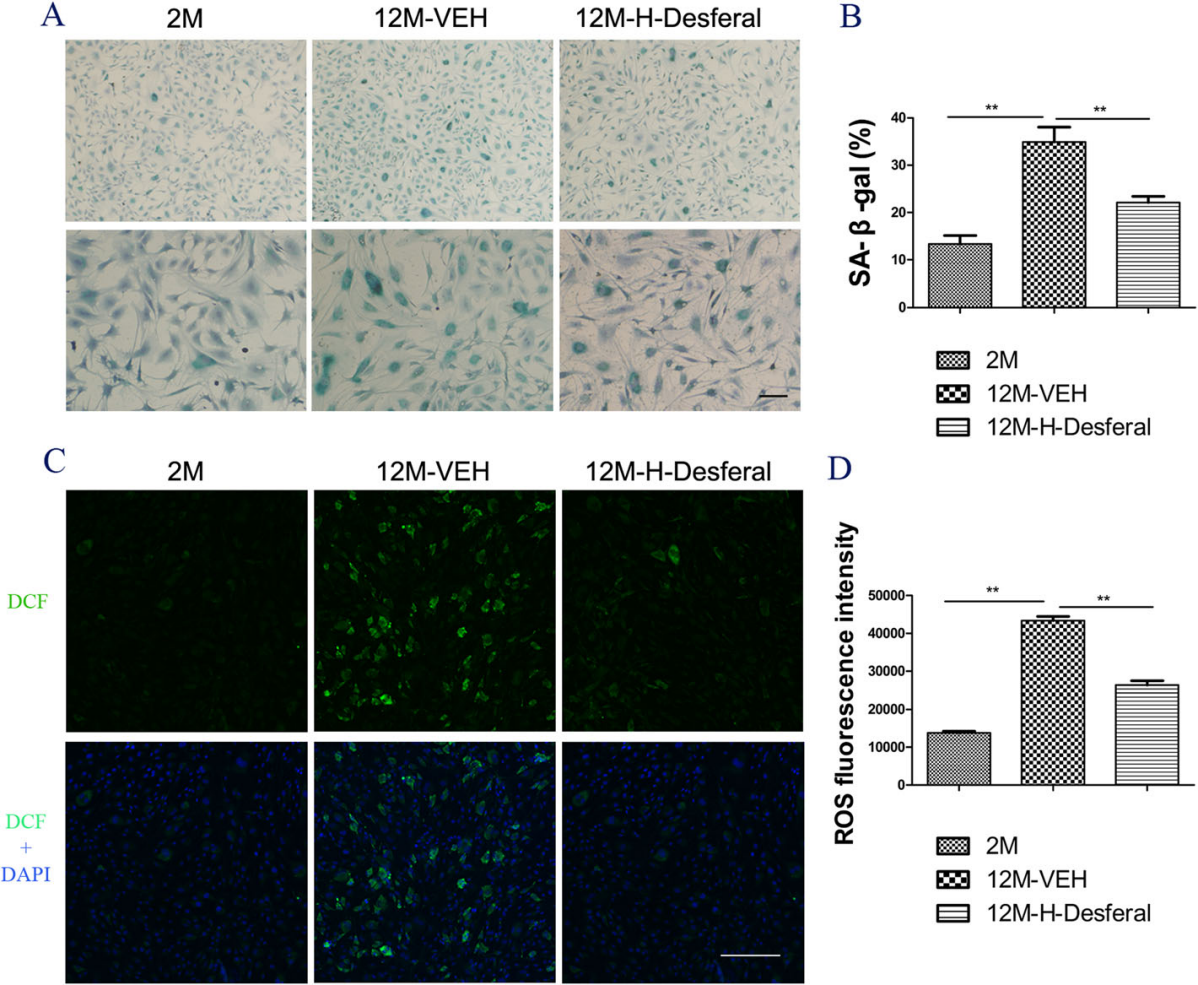
Changes of cell senescence and ROS in BMSCs from rats with and without Desferal® treatment. **a** Representative images of SA-β-gal staining. Blue stained cells indicates positive for senescence. Bar = 200 μm. **b** Quantitative assessment the percent of SA-β-gal positive cells. The percentage of senescence cell was significantly reduced in BMSCs from the 12M-Desferal® group compared with that from the 12M-VEH group. **p < 0.01. **c** Reactive oxygen species (ROS)-induced fluorescence in BMSCs at cell slides was visualized by confocal microscopy. ROS could react with a fluorescence sensor to produce a fluorescent product named as 2′, 7′-dichlorodihydrofluorescein (DCF), which is proportional to the content of ROS. Bar = 200 μm. **d** ROS quantification of BMSC on a 96-well plate by microplate assays. Decreased intracellular ROS levels were observed in BMSCs from the 12M-H-Desferal® group compared with that from the 12M-VEH group. **p < 0.01

To determine the effects of Desferal® on cell senescence of BMSCs, SA-β-gal staining was performed on BMSCs from rats treated with and without Desferal®. BMSCs from 2M group were set as reference. Results from SA-β-gal staining showed that more positive cells in BMSCs from the 12M-VEH group and SA-β-gal-positive cells decreased after Desferal® treatment (Fig. 5a). Quantification assessment suggested that higher percent of SA-β-gal-positive cells was likely associated with aging while short-term Desferal® treatment significantly alleviated age-related cell senescence (Fig. 5b). Considering that ROS played an important role in cell senescence, the ROS in BMSCs from different groups were further examined. A remarkably increased level of ROS was observed in BMSCs from 12M-VEH rats, while significantly decreased ROS was found in BMSCs from the 12M-Desferal® group (Fig. 5c). Microplate assays for ROS fluorescence intensity also showed that ROS production was dramatically increased in BMSC from the 12M-VEH group and high-level ROS was significantly decreased in BMSC from the 12M-Desferal® group (Fig. 5d). These results indicated that Desferal® is capable of partly mitigating BMSC senescence in middle-aged rats.

### The effects of Desferal® on gene expression of cell senescence and stemness in middle-aged BMSCs

**Fig. 6:**
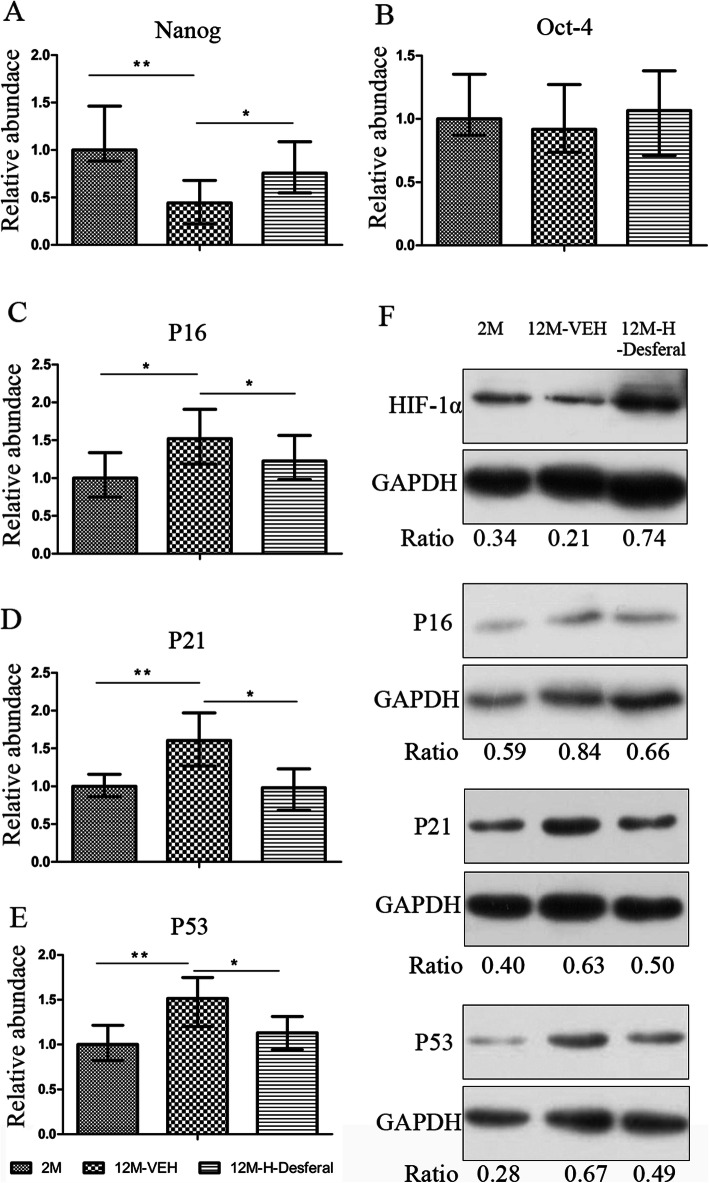
Expression changes of stemness/senescence-associated genes in BMSCs from rats with and without Desferal® treatment. mRNA levels of Nanog (**a**), Oct-4 (**b**), P16 (**c**), P21 (**d**), and P53 (**e**) were analyzed by real-time PCR. These data were drawn from three independent experiments and the results were expressed as mean ± SD. *p < 0.05, **p < 0.01. **f** Western blot results of HIF-1α, P16, P21, and P53. GAPDH was used to as an internal reference

In order to investigate the underlying molecular mechanisms contributed to improved BMSCs properties, we analyzed the expression of stemness and senescence-associated genes. Real-time PCR results for the relative expression of Nanog, OCT-4, P16, P21, and P53 in each group are shown in Fig. 6. We found that notably difference of Nanog expression was observed between the 12M-VEH and 2M groups, and the downregulated expression of Nanog was partial reversed in BMSCs obtained from 12M-Desferal® group (Fig. 6a). No statistical difference of Oct-4 expression was found (Fig. 6b). As shown in Fig. 6c–e, there were significant upregulation of P16, P21, and P53 in BMSCs from 12M-VEH, and markedly downregulation after Desferal® injection. Results from Western blot showed that HIF-1α expression in BMSCs from 12M-Desferal® group was increased compared with those from 12M-VEH group (Fig. 6f). In addition, the increased expression of P16, P21, and P53 was observed in the BMSCs from the 12M-VEH group, and their expression was decreased in the BMSCs from the 12M-Desferal® group (Fig. 6f). Based on these results, we proposed that expression changes of these genes may be greatly responsible for the effect of Desferal® on recovering the stemness and inhibiting the senescence of BMSCs from middle-aged rats.

## Discussion

Osteoporosis and osteoporotic fractures are enormous public health problems, and their incidence and harmfulness increase significantly with the aging of the population. It is well-known that aging is related to decreased bone mass and dysfunctions of stromal cells in the bone marrow. There have been great efforts in exploring how to delay or rejuvenate age-related skeleton changes and senescence of stromal cells [18]. In the current study, we found that long-term injection of Desferal® could reduce age-related bone loss and short-term injection of Desferal® could partially rejuvenate the senescence of BMSCs.

The main reason that we used two different treatment protocols is that the effects on bone loss and BMSC senescence are two relatively independent topics that we investigated in our study. According to previous studies, bone formation and turnover are relatively slow processes. Therefore, we chose to measure the changes in bone mass only after 8 weeks of drug treatment and use this measure as long-term effect of the Desferal® injection. By contrast, the change of BMSCs was reported after 4 day’s short-term treatment [19], and the effect of Desferal® injection on the soft tissue reconstruction has been observed as early as 10 days [20]. Therefore, short-term administration (10-day daily injection) was used to explore the effect of Desferal® on the characteristic of BMSCs by action on the bone marrow microenvironment.

Our results demonstrated that bone mass has significantly increased in middle-aged rats received Desferal® for 8 weeks compared with those without Desferal® treatment, as evidenced by micro-CT and histological analysis. Since Desferal® is a clinically available drug, not an experimental agent, our results revealed the great potential and immediate availability of Desferal® in treating a new indication in the aging-induced osteoporosis. Previous studies have consistently shown the therapeutic effects of DFO on bone loss in a variety of conditions [21–23]. Accelerated bone regeneration was also observed in the bone defect model when treated with DFO locally or systemically [24, 25]. In an ovariectomy (OVX)-induced osteoporotic mouse model, DFO administration prevented bone loss to a certain extent and maintained the microstructure of bone trabeculae [26]. Manipulation of hypoxia signal by genetic or pharmacological methods could stimulate bone formation by coupling osteogenesis and angiogenesis [27]. It has been reported that DFO treatment increased the number of type H vessel and osteoprogenitors by targeting HIF-1α signal [28]. However, to our knowledge, our study is the first time to use an aging-related model to evaluate the bone effects of Desferal®. In this study, we set a low-dose (20 mg/kg) and high-dose (60 mg/kg) Desferal® treatment group according to the dose range described in the drug instructions and found that stimulation of Desferal® on bone was in a dose-dependent manner and no significant effect in the low-dose group. For the underlying mechanism, we proposed that, in addition to acting on the coupling of osteogenesis and angiogenesis, the rejuvenation of endogenous BMSC senescence may also partly contribute to the effects of Desferal® on aging-related osteopenia,.

Aging played an important role in a decline in endogenous stem cell activity including MSC proliferation rate and osteogenic capacity [18]. The change of age-related molecules in the bone marrow niche is one of the driving factors that affecting BMSC osteogenic differentiation [29]. In addition, bone extracellular matrix also worked as important stem cell niche to regulate stem cell behavior, and matrix from young donors could rejuvenate aged MSC [7]. Therefore, microenvironment modulation is a feasible strategy for mitigating and/or rejuvenating senescent BMSC. Desferal®, a hypoxia-mimetic agent, has been shown to upregulate HIF-1α expression by competing for endogenous iron (II) to inhibit PHD activity [30, 31]. HIF-1α is a primary transcriptional factor responding to hypoxia and promotes BMSCs to express osteogenic and angiogenic factors. In addition, hypoxic condition is important for maintaining the normal physiology of BMSCs and downregulate the expression of some senescence markers in vitro [32, 33]. It has been reported that hypoxia inhibit BMSC senescence and maintains cell properties by HIF-TWIST signal [34]. In this study, we confirmed the increased expression of HIF-1α in BMSCs from the 12M-Desferal® group. We also found that Desferal®-treated BMSCs exhibited a decline in the ROS level and expression of senescence-associated genes P16, P21, and P53. Therefore, we proposed that activation of HIF-1α by Desferal® would revise the BMSCs microenvironment and increase cell properties of BMSCs. However, the underlying molecular mechanism requires further study.

Accumulating evidences have shown that a decline of BMSC cellularity, colony formation capacity, and growth kinetics with increased donor age [35, 36]. In the present study, we also confirmed that age-related dysfunction of BMSCs from middle-aged donor compared with that from 2-month-old donor. Most importantly, our results, for the first time, showed that BMSCs isolated from Desferal®-treated middle-aged donors exhibited increased cell growth, in forms of proliferation and colony formation ability, and decreased adipogenic ability, ROS generation, and the number of β-gal-positive cells. We also found that injection of Desferal®, a hypoxia-mimetic agent, favored the expression of stemness markers Nanog in the BMSCs compared with that from untreated middle-aged rats. In addition, we showed that the recovery of BMSC dysfunction from Desferal®-treated middle-aged rats was usually accompanied by reduced expression of senescence-associated genes P16, P21, and P53. These results suggest that short-term Desferal® administration could mitigate aging-induced senescence of BMSCs to a certain extent. In line with our findings, previous studies have suggested the critical role of hypoxia treatment in the enhanced cell proliferation rate, retention of stemness, inhibition of senescence, and increased differentiation ability compared to normoxia [33]. It has been demonstrated that stabilized HIF-1α could enhance the expression of Oct-4 and Nanog [37]. Our findings provide a potential method by targeting the HIF signal with Desferal® to partly reverse age-related handicaps for the BMSC population.

Our results suggested that Desferal® treatment could mitigate the senescence of BMSCs possibly through acting on bone microenvironment. Consistent with our findings, it has reported that a brief neonatal hypoxia may confer higher resistance to senescence in aged male rats [38]. By contrast, Xing et al. reported that chronic hypoxia predisposes BMSCs to premature senescence [39]. We speculate that the different effects of hypoxia on aging may be related to the exposure period of hypoxia and different cell types. Different hypoxia treatment methods and the anti-senescent effect of hypoxia in different tissues need to be further studied. We showed Desferal® has a significant effect on age-related osteopenia and rejuvenates middle-aged BMSCs, but there are still some limitations in our study. For example, a base-line control group was not included our study, therefore, the scale of the drug effect on bone mass was not fully explored. In addition, the BMSC used in this study is a mixture of various cell subsets which may introduce more cell-dependent variations in our results than using c-kit-positive cells. Moreover, this study only examined the effects of Desferal® on the aging of bone tissue and the senescence of BMSCs ex vivo. Whether it has anti-senescent effects in other tissues remains unclear and is worth further investigation.

## Conclusion

In summary, we have demonstrated that Desferal® could reduce age-related bone loss through activating HIF-1α pathway and rejuvenating aged BMSCs, with the comparative measurements in cell proliferation, osteogenic differentiation, senescence, and stemness. Our study provides proof-of-concept evidence for the potential role of clinical drugs targeting hypoxia signals in managing age-associated bone loss and developing BMSC-based cell therapies.

## Supplementary Information

**Additional file 1: Figure S1.** Comparative adipocytes in mid-aged bone by H&E staining and Oil red O staining.

**Additional file 2: Figure S2.** Quantification of BMSCs colonies with more than 50 cells. *p < 0.05,**p < 0.01.

**Additional file 3: Figure S3.** Quantitative assessment the percent of Oil red O staining cells. **p < 0.01, ***p < 0.001.

## Data Availability

The datasets supporting the results of this article are included in the article.

## Abbreviations

BMSCs: Bone marrow stromal cells
HIF-1α: Hypoxia-inducible factors-1α
DFO: Deferoxamine
SD: Sprague-Dawley
CO_2_: Carbon dioxide
PHDs: Prolyl hydroxylases
FDA: Food and Drug Administration
Micro-CT: Micro-computed tomography
BV/TV: Bone volume/tissue volume
Tb.Th: Trabecular thickness
Tb.N: Trabecular number
Tb.Sp: Trabecular separation
SMI: Structural model index
Conn.D: Connectivity density
EDTA: Ethylenediaminetetraacetic acid
HE: Hematoxylin/eosin
FBS: Fetal bovine serum
CCK-8: Cell-Counting Kit-8
OD: Optical density
ARS: Alizarin red S
DCFH-DA: 2′,7′-dichlorofluorescein diacetate
PRP: Platelet-rich plasma
SA-β-gal: Senescence-associated β-galactosidase
ROS: Reactive oxygen species
