# Supplementary figures and images for: Intraperitoneal injection of Desferal® alleviated the age-related bone loss and senescence of bone marrow stromal cells in rats
Source: Stem Cell Res Ther. 2021 Jan 7;12:45. doi: 10.1186/s13287-020-02112-9 (PMC7791659; doi:10.1186/s13287-020-02112-9)

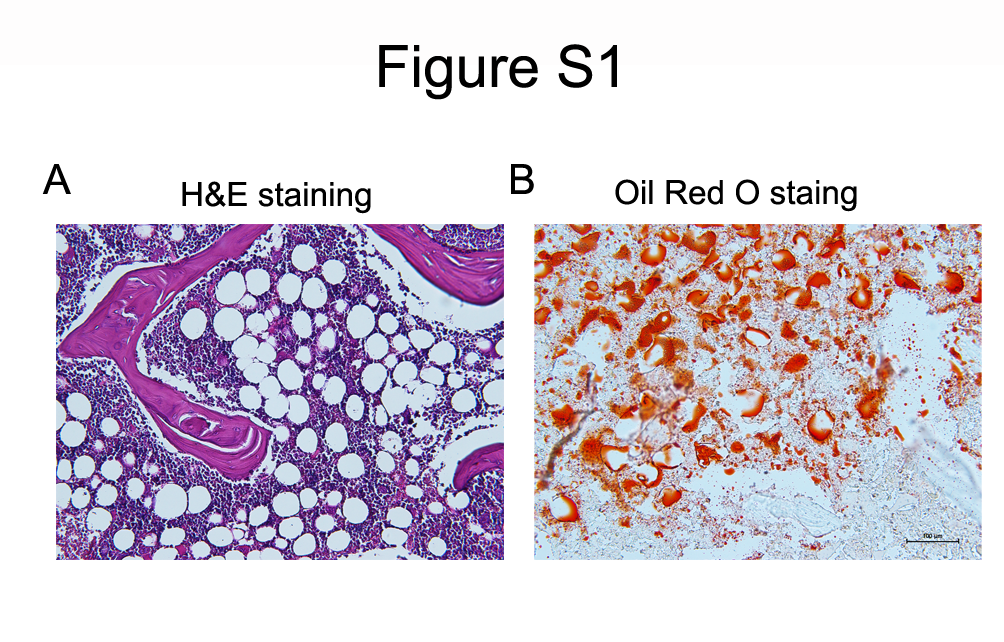

Supplement: Supplementary file 1 — Additional file 1: Figure S1. Comparative adipocytes in mid-aged bone by H&E staining and Oil red O staining. [file 13287_2020_2112_MOESM1_ESM.tif]

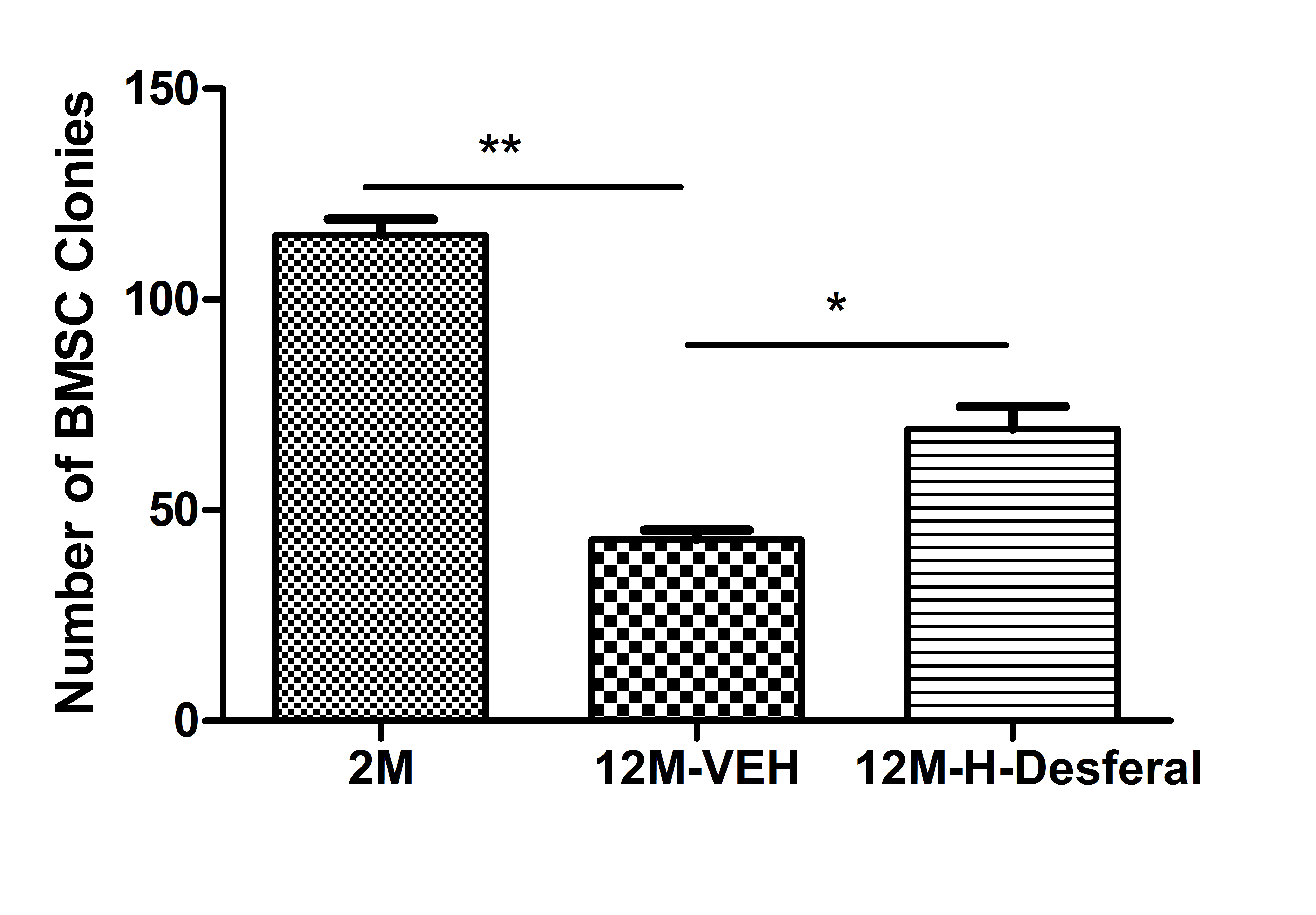

Supplement: Supplementary file 2 — Additional file 2: Figure S2. Quantification of BMSCs colonies with more than 50 cells. *p < 0.05,**p < 0.01. [file 13287_2020_2112_MOESM2_ESM.tif]

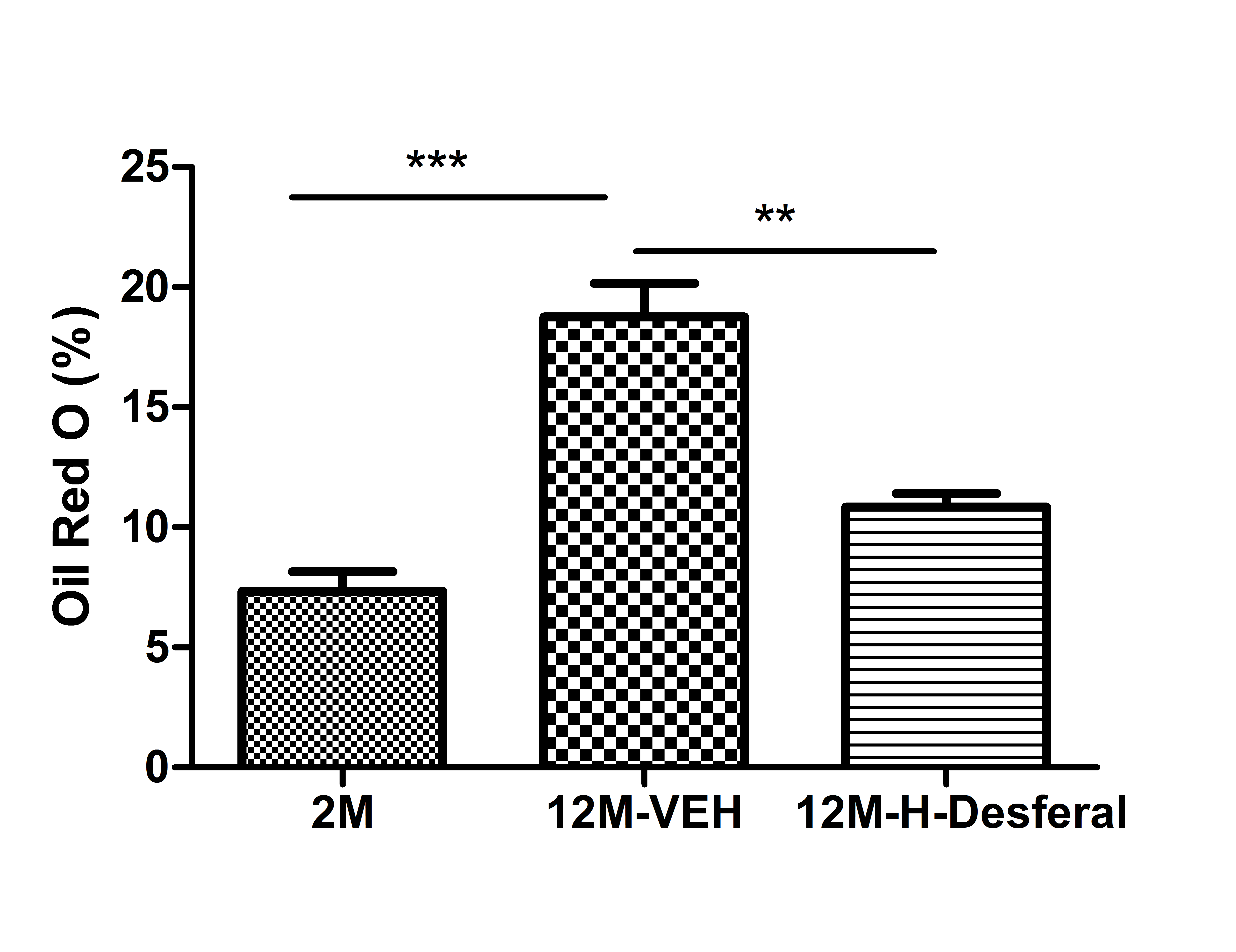

Supplement: Supplementary file 3 — Additional file 3: Figure S3. Quantitative assessment the percent of Oil red O staining cells. **p < 0.01, ***p < 0.001. [file 13287_2020_2112_MOESM3_ESM.tif]
